# Diphen­yl[(phenyl­sulfan­yl)meth­yl]-λ^5^-phosphane­thione

**DOI:** 10.1107/S1600536814004292

**Published:** 2014-02-28

**Authors:** Viktoria H. Gessner

**Affiliations:** a Institut fuer Anorganische Chemie, Julius-Maximilians-Universitaet Würzburg, Am Hubland, 97074 Würzburg, Germany

## Abstract

The title compound, C_19_H_17_PS_2_, results from the direct deprotonation of di­phenyl­methyl­phosphine sulfide and subsequent reaction with diphenyl di­sulfide. The C—P and C—S bond lengths of 1.8242 (18) and 1.8009 (18) Å, respectively, of the central P—C—S linkage are comparable to those found in the sulfonyl analogue, but are considerably longer than those reported for the dimetallated sulfonyl compound. The dihedral angle between the benzene rings of the di­phenyl­methyl moiety is 69.46 (7)°. No distinct inter­molecular inter­actions are present in the crystal structure.

## Related literature   

For the sulfonyl and dimetallated sulfonyl analogues, see: Schröter & Gessner (2012 ▶). For background to precursors for dili­thio methandiides and their carbene complexes, see: Becker & Gessner (2014*a* ▶,*b* ▶); Cantat *et al.* (2006 ▶, 2008 ▶); Cavell *et al.* (2001 ▶); Cooper *et al.* (2010 ▶); Gessner (2012 ▶); Gessner *et al.* (2013 ▶); Harder (2011 ▶); Kasani *et al.* (1999 ▶); Liddle *et al.* (2011 ▶); Ong *et al.* (1999 ▶).
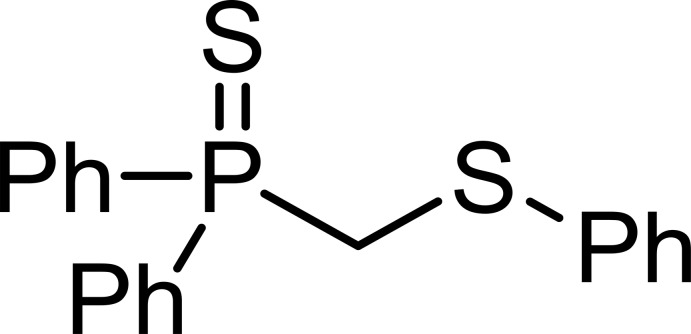


## Experimental   

### 

#### Crystal data  

C_19_H_17_PS_2_
*M* *_r_* = 340.42
Monoclinic, 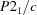

*a* = 9.3748 (13) Å
*b* = 18.598 (3) Å
*c* = 10.0941 (14) Å
β = 101.044 (2)°
*V* = 1727.3 (4) Å^3^
*Z* = 4
Mo *K*α radiation
μ = 0.39 mm^−1^
*T* = 173 K
0.38 × 0.18 × 0.15 mm

#### Data collection  

Bruker APEX CCD diffractometer
Absorption correction: multi-scan (*SADABS*; Bruker, 1999 ▶) *T*_min_ = 0.953, *T*_max_ = 0.979
10734 measured reflections
3033 independent reflections
2663 reflections with *I* > 2σ(*I*)
*R*_int_ = 0.041

#### Refinement  

*R*[*F* ^2^ > 2σ(*F* ^2^)] = 0.034
*wR*(*F* ^2^) = 0.091
*S* = 1.05
3033 reflections
199 parameters
H-atom parameters constrained
Δρ_max_ = 0.31 e Å^−3^
Δρ_min_ = −0.23 e Å^−3^

### 

Data collection: *SMART* (Bruker, 2001 ▶); cell refinement: *SAINT-Plus* (Bruker, 1999 ▶); data reduction: *SAINT-Plus*; program(s) used to solve structure: *SHELXS97* (Sheldrick, 2008 ▶); program(s) used to refine structure: *SHELXL97* (Sheldrick, 2008 ▶); molecular graphics: *ORTEP-3 for Windows* (Farrugia, 2012 ▶); software used to prepare material for publication: *SHELXL97*.

## Supplementary Material

Crystal structure: contains datablock(s) I, global. DOI: 10.1107/S1600536814004292/wm5008sup1.cif

Structure factors: contains datablock(s) I. DOI: 10.1107/S1600536814004292/wm5008Isup2.hkl

Supporting information file. DOI: 10.1107/S1600536814004292/wm5008Isup3.cml

CCDC reference: 988671

Additional supporting information:  crystallographic information; 3D view; checkCIF report

## Figures and Tables

**Fig. 1.**
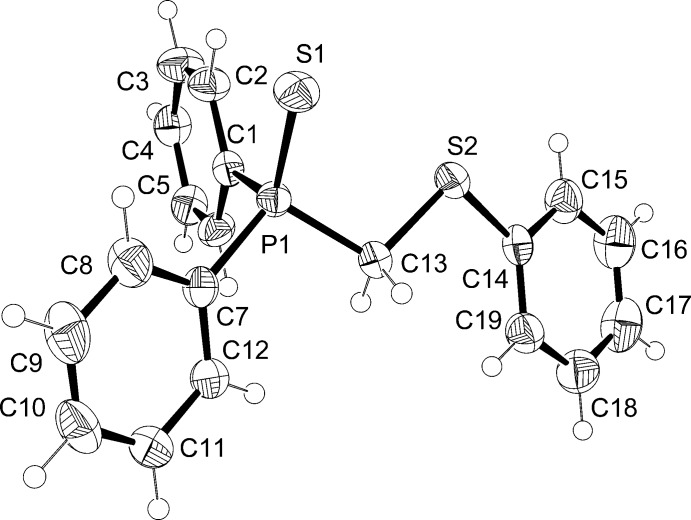
The molecular structure of the title compound. Displacement parameters are drawn at the 50% probability level.

## supplementary crystallographic information

### 1. Comment

Methylene compounds with two anion-stabilizing substituents, such as phosphonium or sulfonyl moieties, have found special interest as precursors for the corresponding dimetallated methandiides (Kasani *et al.*, 1999; Ong *et al.*, 1999; Cantat *et al.*, 2006; Cooper *et al.*, 2010). These dianions were found to be excellent ligands for the preparation of carbene complexes exhibiting a unique electronic structure (Gessner *et al.*, 2013). They allowed the synthesis of a variety of different complexes with early and late transition metals, but also with lanthanides and actinides (Cavell *et al.*, 2001; Cantat *et al.*, 2008; Harder, 2011; Liddle *et al.*, 2011).

As part of our studies on the synthesis of novel methandiides for the preparation of carbene complexes, we have developed a ligand and its dianionic analogue with a thiophosphoryl and sulfonyl sidearm (Schröter & Gessner, 2012). Thereby, the synthesis of a protonated precursor is best achieved *via* a two-step synthesis, with the first step being the lithiation of diphenylmethylphosphine sulfide and its reaction with diphenyl disulfide (Becker & Gessner, 2014*a*,*b*). This procedure furnishes the title compound, C_19_H_17_PS_2_, in good yield. Oxidation of the sulfide to the sulfone gives way to the final ligand (Gessner, 2012).

The bond lengths and angles in the title compound are comparable to the sulfonyl analogue, but deviate considerably from the dimetallated compound (Schröter & Gessner, 2012). These differences are most pronounced in the P—C—S backbone. While the title compound features C—P and C—S distances of 1.8242 (18) and 1.8009 (18) Å, respectively, the sulfonyl substituted dianion shows C—P_av_ distances shortened by ≈ 7% [1.710 (4) Å] and C—S distances shortened by ≈ 11% [1.614 (3) Å] (Schröter & Gessner, 2012). Additionally, the P—C—S angle experiences a widening from 107.7 (1)° in the title compound to 121.4 (2)° in the methandiide. This is the result of a change in the hybridization of the central carbon atom from *sp*^3^ in the title compound to *sp*^2^ in the methandiide.

No distinct intermolecular interactions (such as C—H···S interactions) are present in the crystal structure of the title compound.

### 2. Experimental

4.00 g (17.2 mmol) diphenylmethylphosphane sulfide were dissolved in 50 ml THF and 11.1 ml (17.2 mmol) *n*-butyllithium (1.55 *M* solution in hexane) were added drop-wise at 195 K. The mixture was allowed to warm to room temperatureand stirred for additional 3 h. After cooling to 195 K 3.76 g (17.2 mmol) diphenyldisulfide dissolved in 30 ml THF were added. The mixture was stirred overnight, quenchedby the addition of 50 ml water and extracted three times with 40 ml diethyl ether. After drying over sodium sulfate, the solvent was removed *in vacuo* and the residue purified by bulb-to-bulb distillation (oven temperature 473–483 K, 1x10^-3^ mbar). The title compound was obtained as a yellowish oil, which solidified after a couple of days (4.21 g, 12.3 mmol; 72%). ^1^H NMR: (300.1 MHz, CDCl_3_): δ =3.92 (d, ^2^*J*_PH_ = 9.09 Hz, 2H; PC*H*_2_S),7.16–7.19 (m, 3H; C*H*_SPh,_*_meta_*_/_*_para_*), 7.23–7.27 (m, 2H; C*H*_SPh,_*_ortho_*),7.40–7.47 (m, 6H; C*H*_PPh,_*_meta_*_/_*_para_*), 7.72–7.79 (m, 4H; C*H*_PPh,_*_ortho_*).^13^C{^1^H} NMR: (75.5 MHz, CDCl_3_): δ = 38.5 (d, ^1^*J*_PC_ =52.25 Hz; P*C*H_2_S), 127.1 (*C*_SPh,_*_ortho_*), 128.6(d, ^3^*J*_PC_ = 12.33 Hz; *C*_PPh,_*_meta_*),129.0 (*C*_SPh,_*_meta_*), 130.4 (*C*_SPh,_*_para_*), 131.0(d, _1_*J*_PC_ = 82.59 Hz; *C*_PPh,_*_ipso_*),131.6 (d, ^4^*J*_PC_ = 10.24 Hz; *C*_PPh,_*_ortho_*),131.9 (d, ^3^*J*_PC_ = 2.90 Hz, *C*_PPh,_*_para_*),135.5 (d, ^2^*J*_PC_ = 5.78 Hz; *C*_SPh,_*_ipso_*).^31^P{^1^H} NMR: (122.0 MHz, CDCl_3_): δ =40.6. Anal. Calcd for C_19_H_17_PS: C, 67.03; H, 5.03; S, 18.84; found: C, 66.80; H, 5.00; S, 19.09; GC—MS(ESI): tR = 17.18 min [353 K (2 min) -10 K min-1 – 553 K (5 min)]; *m/z* (%): 340 (36) (*M*+), 217 (100) {[Ph_2_PS]+}, 123 (46) {[CH_2_SPh]+}, 139 (81) {[C_6_H_4_PS]+}.

### 3. Refinement

The H atoms were refined on a riding model approximation in their ideal geometric positions with C—H = 0.95 Å for C–H(aromatic) and 0.99 for CH_2_ atoms, respectively, with *U*_iso_(H) = 1.2*U*_eq_(C).

### Crystal data

**Table:**

C_19_H_17_PS_2_	*F*(000) = 712
*M**_r_* = 340.42	*D*_x_ = 1.309 Mg m^−^^3^
Monoclinic, *P*2_1_/*c*	Mo *K*α radiation, λ = 0.71073 Å
Hall symbol: -P 2ybc	Cell parameters from 4352 reflections
*a* = 9.3748 (13) Å	θ = 2.2–25°
*b* = 18.598 (3) Å	µ = 0.39 mm^−^^1^
*c* = 10.0941 (14) Å	*T* = 173 K
β = 101.044 (2)°	Block, colourless
*V* = 1727.3 (4) Å^3^	0.38 × 0.18 × 0.15 mm
*Z* = 4	

### Data collection

**Table:**

Bruker APEX CCD diffractometer	3033 independent reflections
Radiation source: fine-focus sealed tube	2663 reflections with *I* > 2σ(*I*)
Graphite monochromator	*R*_int_ = 0.041
ω–scans	θ_max_ = 25°, θ_min_ = 2.2°
Absorption correction: multi-scan (*SADABS*; Bruker, 1999)	*h* = −11→7
*T*_min_ = 0.953, *T*_max_ = 0.979	*k* = −22→22
10734 measured reflections	*l* = −12→11

### Refinement

**Table:**

Refinement on *F*^2^	Primary atom site location: structure-invariant direct methods
Least-squares matrix: full	Secondary atom site location: difference Fourier map
*R*[*F*^2^ > 2σ(*F*^2^)] = 0.034	Hydrogen site location: inferred from neighbouring sites
*wR*(*F*^2^) = 0.091	H-atom parameters constrained
*S* = 1.05	*w* = 1/[σ^2^(*F*_o_^2^) + (0.0403*P*)^2^ + 0.5374*P*] where *P* = (*F*_o_^2^ + 2*F*_c_^2^)/3
3033 reflections	(Δ/σ)_max_ = 0.007
199 parameters	Δρ_max_ = 0.31 e Å^−^^3^
0 restraints	Δρ_min_ = −0.23 e Å^−^^3^
0 constraints	

### Special details

**Table:**

Geometry. All e.s.d.'s (except the e.s.d. in the dihedral angle between two l.s. planes) are estimated using the full covariance matrix. The cell e.s.d.'s are taken into account individually in the estimation of e.s.d.'s in distances, angles and torsion angles; correlations between e.s.d.'s in cell parameters are only used when they are defined by crystal symmetry. An approximate (isotropic) treatment of cell e.s.d.'s is used for estimating e.s.d.'s involving l.s. planes.
Refinement. Refinement of *F*^2^ against ALL reflections. The weighted *R*-factor *wR* and goodness of fit *S* are based on *F*^2^, conventional *R*-factors *R* are based on *F*, with *F* set to zero for negative *F*^2^. The threshold expression of *F*^2^ > σ(*F*^2^) is used only for calculating *R*-factors(gt) *etc*. and is not relevant to the choice of reflections for refinement. *R*-factors based on *F*^2^ are statistically about twice as large as those based on *F*, and *R*-factors based on ALL data will be even larger.

### Fractional atomic coordinates and isotropic or equivalent isotropic displacement parameters (Å^2^)

**Table:**

	*x*	*y*	*z*	*U*_iso_*/*U*_eq_	
C1	0.52214 (19)	0.08028 (9)	0.30067 (17)	0.0288 (4)	
C2	0.5712 (2)	0.02245 (10)	0.23596 (19)	0.0380 (4)	
H2	0.535	0.0147	0.1425	0.046*	
C3	0.6729 (2)	−0.02428 (11)	0.3069 (2)	0.0472 (5)	
H3	0.7053	−0.0643	0.2623	0.057*	
C4	0.7274 (2)	−0.01291 (10)	0.4421 (2)	0.0413 (5)	
H4	0.7987	−0.0445	0.49	0.05*	
C5	0.6782 (2)	0.04426 (10)	0.50795 (19)	0.0397 (5)	
H5	0.7153	0.0518	0.6013	0.048*	
C6	0.5750 (2)	0.09060 (10)	0.43818 (18)	0.0356 (4)	
H6	0.5401	0.1295	0.484	0.043*	
C7	0.2351 (2)	0.14772 (9)	0.28387 (18)	0.0311 (4)	
C8	0.1148 (2)	0.10634 (12)	0.2316 (2)	0.0463 (5)	
H8	0.1162	0.0763	0.1558	0.056*	
C9	−0.0079 (2)	0.10857 (13)	0.2896 (2)	0.0551 (6)	
H9	−0.0897	0.0796	0.2539	0.066*	
C10	−0.0118 (2)	0.15236 (13)	0.3978 (2)	0.0510 (6)	
H10	−0.0961	0.1538	0.4372	0.061*	
C11	0.1064 (2)	0.19424 (11)	0.4494 (2)	0.0487 (5)	
H11	0.1031	0.225	0.5239	0.058*	
C12	0.2297 (2)	0.19198 (10)	0.3942 (2)	0.0402 (5)	
H12	0.3114	0.2207	0.4314	0.048*	
C13	0.4787 (2)	0.23097 (9)	0.24179 (18)	0.0322 (4)	
H13A	0.4074	0.2696	0.2109	0.039*	
H13B	0.5143	0.2366	0.3401	0.039*	
C14	0.7380 (2)	0.30474 (9)	0.24404 (18)	0.0332 (4)	
C15	0.8792 (2)	0.30812 (11)	0.2215 (2)	0.0467 (5)	
H15	0.9118	0.2738	0.1642	0.056*	
C16	0.9721 (2)	0.36083 (13)	0.2815 (3)	0.0568 (6)	
H16	1.0679	0.3635	0.264	0.068*	
C17	0.9273 (3)	0.40970 (13)	0.3668 (2)	0.0573 (6)	
H17	0.9923	0.4458	0.4087	0.069*	
C18	0.7882 (3)	0.40641 (12)	0.3914 (2)	0.0534 (6)	
H18	0.7574	0.4402	0.4505	0.064*	
C19	0.6925 (2)	0.35395 (11)	0.3302 (2)	0.0432 (5)	
H19	0.5964	0.3518	0.3473	0.052*	
P1	0.39369 (5)	0.14308 (2)	0.20578 (4)	0.02841 (14)	
S1	0.34680 (6)	0.12132 (3)	0.01308 (5)	0.03941 (16)	
S2	0.62814 (5)	0.23650 (3)	0.15364 (5)	0.03730 (15)	

### Atomic displacement parameters (Å^2^)

**Table:**

	*U*^11^	*U*^22^	*U*^33^	*U*^12^	*U*^13^	*U*^23^
C1	0.0287 (9)	0.0300 (9)	0.0276 (9)	−0.0035 (7)	0.0050 (7)	0.0025 (7)
C2	0.0430 (12)	0.0405 (10)	0.0295 (9)	0.0046 (9)	0.0047 (9)	−0.0024 (8)
C3	0.0533 (13)	0.0452 (11)	0.0428 (12)	0.0134 (10)	0.0090 (10)	−0.0047 (9)
C4	0.0389 (11)	0.0410 (11)	0.0422 (11)	0.0053 (9)	0.0031 (9)	0.0097 (9)
C5	0.0447 (12)	0.0409 (11)	0.0300 (10)	−0.0054 (9)	−0.0017 (9)	0.0033 (8)
C6	0.0440 (12)	0.0315 (9)	0.0303 (9)	−0.0023 (8)	0.0044 (9)	−0.0036 (7)
C7	0.0284 (10)	0.0311 (9)	0.0327 (9)	−0.0017 (7)	0.0035 (8)	0.0038 (7)
C8	0.0412 (12)	0.0577 (13)	0.0393 (11)	−0.0143 (10)	0.0055 (10)	−0.0050 (9)
C9	0.0346 (12)	0.0731 (16)	0.0558 (14)	−0.0193 (11)	0.0038 (11)	0.0046 (12)
C10	0.0346 (12)	0.0627 (14)	0.0595 (14)	0.0025 (10)	0.0187 (11)	0.0135 (11)
C11	0.0469 (13)	0.0447 (11)	0.0607 (13)	0.0000 (10)	0.0259 (11)	−0.0029 (10)
C12	0.0376 (11)	0.0376 (10)	0.0485 (11)	−0.0076 (9)	0.0158 (9)	−0.0062 (9)
C13	0.0333 (10)	0.0323 (9)	0.0326 (9)	−0.0035 (7)	0.0102 (8)	0.0012 (7)
C14	0.0336 (10)	0.0336 (9)	0.0322 (9)	−0.0029 (8)	0.0054 (8)	0.0091 (7)
C15	0.0374 (12)	0.0456 (12)	0.0589 (13)	−0.0007 (9)	0.0140 (10)	0.0068 (10)
C16	0.0345 (12)	0.0594 (14)	0.0737 (16)	−0.0078 (10)	0.0032 (12)	0.0103 (12)
C17	0.0520 (15)	0.0561 (14)	0.0552 (14)	−0.0188 (11)	−0.0111 (12)	0.0049 (11)
C18	0.0675 (16)	0.0501 (13)	0.0414 (12)	−0.0115 (11)	0.0075 (11)	−0.0078 (10)
C19	0.0445 (12)	0.0440 (11)	0.0430 (11)	−0.0064 (9)	0.0132 (10)	−0.0019 (9)
P1	0.0297 (3)	0.0295 (2)	0.0257 (2)	−0.00220 (18)	0.0045 (2)	−0.00026 (17)
S1	0.0459 (3)	0.0451 (3)	0.0251 (3)	0.0004 (2)	0.0013 (2)	−0.00118 (19)
S2	0.0409 (3)	0.0377 (3)	0.0369 (3)	−0.0078 (2)	0.0167 (2)	−0.00314 (19)

### Geometric parameters (Å, º)

**Table:**

C1—C2	1.382 (3)	C11—C12	1.377 (3)
C1—C6	1.395 (2)	C11—H11	0.95
C1—P1	1.8138 (18)	C12—H12	0.95
C2—C3	1.384 (3)	C13—S2	1.8009 (18)
C2—H2	0.95	C13—P1	1.8242 (18)
C3—C4	1.379 (3)	C13—H13A	0.99
C3—H3	0.95	C13—H13B	0.99
C4—C5	1.379 (3)	C14—C19	1.385 (3)
C4—H4	0.95	C14—C15	1.387 (3)
C5—C6	1.384 (3)	C14—S2	1.7729 (19)
C5—H5	0.95	C15—C16	1.373 (3)
C6—H6	0.95	C15—H15	0.95
C7—C8	1.384 (3)	C16—C17	1.371 (3)
C7—C12	1.393 (3)	C16—H16	0.95
C7—P1	1.8137 (18)	C17—C18	1.375 (3)
C8—C9	1.388 (3)	C17—H17	0.95
C8—H8	0.95	C18—C19	1.387 (3)
C9—C10	1.369 (3)	C18—H18	0.95
C9—H9	0.95	C19—H19	0.95
C10—C11	1.373 (3)	P1—S1	1.9527 (7)
C10—H10	0.95		
			
C2—C1—C6	119.37 (17)	C11—C12—H12	119.9
C2—C1—P1	119.93 (14)	C7—C12—H12	119.9
C6—C1—P1	120.68 (13)	S2—C13—P1	107.72 (9)
C1—C2—C3	120.18 (17)	S2—C13—H13A	110.2
C1—C2—H2	119.9	P1—C13—H13A	110.2
C3—C2—H2	119.9	S2—C13—H13B	110.2
C4—C3—C2	120.23 (19)	P1—C13—H13B	110.2
C4—C3—H3	119.9	H13A—C13—H13B	108.5
C2—C3—H3	119.9	C19—C14—C15	119.44 (18)
C5—C4—C3	120.05 (18)	C19—C14—S2	125.31 (15)
C5—C4—H4	120	C15—C14—S2	115.23 (15)
C3—C4—H4	120	C16—C15—C14	120.3 (2)
C4—C5—C6	120.05 (17)	C16—C15—H15	119.9
C4—C5—H5	120	C14—C15—H15	119.9
C6—C5—H5	120	C17—C16—C15	120.4 (2)
C5—C6—C1	120.09 (17)	C17—C16—H16	119.8
C5—C6—H6	120	C15—C16—H16	119.8
C1—C6—H6	120	C16—C17—C18	119.9 (2)
C8—C7—C12	118.81 (18)	C16—C17—H17	120
C8—C7—P1	118.95 (15)	C18—C17—H17	120
C12—C7—P1	122.23 (14)	C17—C18—C19	120.4 (2)
C7—C8—C9	120.2 (2)	C17—C18—H18	119.8
C7—C8—H8	119.9	C19—C18—H18	119.8
C9—C8—H8	119.9	C14—C19—C18	119.6 (2)
C10—C9—C8	120.3 (2)	C14—C19—H19	120.2
C10—C9—H9	119.8	C18—C19—H19	120.2
C8—C9—H9	119.8	C7—P1—C1	108.53 (8)
C9—C10—C11	119.9 (2)	C7—P1—C13	103.53 (8)
C9—C10—H10	120.1	C1—P1—C13	104.55 (8)
C11—C10—H10	120.1	C7—P1—S1	113.32 (6)
C10—C11—C12	120.5 (2)	C1—P1—S1	113.12 (6)
C10—C11—H11	119.7	C13—P1—S1	112.99 (6)
C12—C11—H11	119.7	C14—S2—C13	102.51 (9)
C11—C12—C7	120.20 (19)		
